# Modularizing speech

**DOI:** 10.3389/fpsyg.2013.00977

**Published:** 2013-12-25

**Authors:** Bryan Gick, Ian Stavness

**Affiliations:** ^1^Department of Linguistics, University of British ColumbiaVancouver, BC, Canada; ^2^Haskins LaboratoriesNew Haven, CT, USA; ^3^Department of Computer Science, University of SaskatchewanSaskatoon, SK, Canada

**Keywords:** speech production, modularization, biomechanics, motor control, neurophysiology, degrees of freedom

The need to reduce the dimensionality of movement systems, and thereby to decrease cognitive load, has long been recognized as a central challenge for theories of motor control (Bernstein, 1967). A large body of work in neurophysiology, biomechanics, and computation has substantiated the view that control of body movements is distributed among a manageable number of degrees of freedom corresponding to neuromuscular *modules* (e.g., Bizzi et al., 1991), or proportionally fixed groupings of muscles (see e.g., Ting et al., 2012 for a recent review). Current work in computational neuroscience provides evidence that the nervous system uses such modules to achieve dimensionality reduction (e.g., Berger et al., 2013). It is our opinion that a fully realized modular approach to speech movement will have a profound impact on models of speech.

In speech-related fields, researchers had begun formulating ideas for modularizing speech movements even prior to Bernstein's influence. Cooper et al. (1958), for instance, in proposing their notion of the “action plan,” described for speech an inventory of muscle activations not unlike Bernstein's “muscle synergies”: “we may hope to describe speech events in terms of a rather limited number of muscle groups… ” (p. 939). Later, Turvey (1977) adopted the term *coordinative structure* to refer to similar neuromuscular groupings. Easton (1972) had first defined coordinative structures as neuromuscular organizations “underlying all volitionally composed movements… activated by a single command,” such that “the CNS [central nervous system] may be said to have at its disposal a library, or set, of these responses” (p. 591). However, Turvey et al. (1978) shifted focus away from neurophysiology, observing that coordinative structures are “formally equivalent” to tasks in control space (1978, p. 566). Subsequent speech researchers have taken this lead, focusing on developing models of control space (e.g., Kelso et al., 1986a; Tourville and Guenther, 2011), with little or no attention given to modeling the neurophysiology of embodied speech.

Meanwhile, researchers in other areas have built a substantial volume of experimental and modeling research around the neuromuscular organization and biomechanics of non-speech movement, including work on complex fine motor systems such as the fingers (e.g., Overduin et al., 2012) and eyes (e.g., Wei et al., 2010). However, speech, along with many other functions of the upper vocal tract, has remained a conspicuous omission from the literature on neuromuscular modularization. This omission may be ascribed at least in part to the relatively greater complexity of both the muscular structures (e.g., Sanders and Mu, 2013) and the multidimensional control space (e.g., Houde and Jordan, 1998; Tremblay et al., 2003; Gick and Derrick, 2009; Ghosh et al., 2010; Perkell, 2012) of speech. Kelso et al. (1986b) describe this position clearly, stating that mapping their control paradigm onto “real” body structures is “not feasible for the speech articulators whose peripheral biomechanics are much more complex (than upper limbs), e.g., the passive tissue properties and muscular forces of the tongue and lips.”

The great majority of evidence for modularization derives from experiments on non-human spinal structures (see Tresch et al., 2002) and from direct recordings of neuromuscular activity using electromyography (see Kutch and Valero-Cuevas, 2012). However, neither of these methods is likely to be as effective for understanding neural control of speech, first because upper airway innervation is predominantly cranial rather than spinal, and second because of the known challenges of experimentally recording comprehensive or even representative neuromuscular activity from EMG, even in less complex tasks than speech (Pittman and Bailey, 2009) and in comparatively less complex neuromuscular systems (Hug, 2011; De Rugy et al., 2013). Because of this, we anticipate that biomechanics will necessarily play a more central role in accessing the modular neuromuscular structures that underlie speech production.

In our view, neuromuscular modules are built specifically to drive body structures that are biomechanically efficacious, enabling them to operate feed-forward, i.e., with little or no central feedback control. This has often been assumed as a premise underlying modularization (e.g., Loeb et al., 2000; d'Avella et al., 2003; Loeb, 2012), but has seldom been tested (see Berniker et al., 2009 for a rare exception), and never applied to speech. Recent advances in modeling speech biomechanics (e.g., Nazari et al., 2011; Stavness et al., 2012a,b) have enabled our group to begin identifying some of the biomechanical properties that we consider to be the hallmarks of speech production modules, most notably pervasive saturation effects that enable feed-forward control of speech structures (Gick et al., in press). At least some of these biomechanically optimized speech production modules correspond well with speech “gestures,” long described as movement-related primitives of speech (e.g., Browman and Goldstein, 1986).

While there remains some controversy around whether these modules are best defined in terms of their neural (e.g., d'Avella and Bizzi, 2005; Safavynia and Ting, 2013), biomechanical (Dominici et al., 2011; Kutch and Valero-Cuevas, 2012), or computational (Todorov, 2004; Diedrichsen et al., 2010; Loeb, 2012; De Rugy et al., 2013) properties, all of these aspects of control will be necessary components of a complete theory (see Bizzi and Cheung, 2013), and at present none of these aspects have been well explored for speech and upper airway control.

Developing a theory of speech production that accords with current work on neuromuscular modularization, we believe, has the potential to link a number of fields and methodologies surrounding a central question in cognitive science, with implications for all aspects of speech research, from phonetics and phonology to the phylogenetic and ontogenetic development of speech. In addition to bringing another complex motor system into the broader discussion of neural modules, modularizing speech at the neuromuscular level promises a major advance for speech models, constituting a “missing link” between speech movement primitives (Ramanarayanan et al., 2013) and newly discovered cortical regions associated with speech production (Bouchard et al., 2013).
